# Limbal reconstruction in uveitic glaucoma patient with exposed Ahmed valve coincident with corneal melting and iris prolapse using multiple corneoscleral allografts

**DOI:** 10.22336/rjo.2024.12

**Published:** 2024

**Authors:** Stylianos Artemios Kandarakis, Leonidas Doumazos, Marios Timpilis, Georgia Karageorgiou, Petros Petrou, Ilias Georgalas

**Affiliations:** * Department of Ophthalmology, National and Kapodistrian University of Athens, 1st University Eye Clinic, G. Gennimatas General Hospital, Athens, Greece

**Keywords:** Ahmed tube, glaucoma, tube exposure, corneoscleral graft

## Abstract

**Aim:** To present a complex case of Ahmed tube exposure 6 months after the implantation associated with corneal melting and iris prolapse, and the surgical reposition that required multiple allografts and limbal reconstruction.

**Methods:** A 60-year-old patient arrived at the emergency room with tube exposure combined with corneal melting and iris prolapse from a previously placed Ahmed valve 6 months prior. Our approach was to use one corneoscleral graft to repair the melted cornea and avoid further iris prolapse and a second scleral graft to cover the repositioned tube. Upon completion of conjunctival dissection, cleaning and deepithelization of the melted cornea and the tube by application of alcohol 100% followed. A new entry point was made for the tube and was covered using an alcohol-preserved scleral allograft and the previous entry point was repaired using a corneoscleral allograft with the corneal aspect restoring the limbus and avoiding further iris protrusion.

**Results:** 6 months follow-up of the patient showed excellent recovery, anatomical restoration, and IOP normalization.

**Conclusion:** Surgical repair of these cases can be very demanding, and requires surgical improvisation and prolonged surgical time. The literature remains very limited on how a surgeon should approach similar cases, which are the crucial tips, and which are the missteps that should be avoided. In this case, we used multiple scleral/corneoscleral allografts in a specific orientation and different sutures to reconstruct the damaged limbal area and restore the anatomy.

**Abbreviations:** VA = Visual Acuity, GDD = Glaucoma Drainage Device, IOP = Intra Ocular Pressure

## Introduction

Ahmed Valve is a shunt device that consists of a silicone tube attached to a valve mechanism on an end plate. It can be considered for use in primary glaucoma surgery, while other indications include previous failure of primary surgery, extensive conjunctival scarring, and likely failure of trabeculectomy [**1**]. Especially in patients with uveitic glaucoma, Ahmed valve implantation offers similar IOP control but an increased rate of success in the first year and, in some studies, longer mean time to failure compared with trabeculectomy with Mitomycin C as the first choice [**2**,**3**]. 

However, tube erosion is a serious situation with higher rates in uveitic glaucoma patients and withholds devastating possibly sight-threatening complications [**4**]. Several authors have proposed different tissues for the graft, including autologous or human donor sclera, pericardium, dura mater, cornea or human cadaveric fascia lata, amniotic membrane, autologous tenon, and expanded polytetrafluoroethylene, the same materials that can be used initially to cover the implanted tube [**1**,**5**]. Because all materials can be used with variable success, the selection is up to the surgeon, who should consider several factors such as surgical experience, affordability, availability, cosmetic appeal, and monitoring of the host site [**6**].

## Case report

**Fig. 1:**
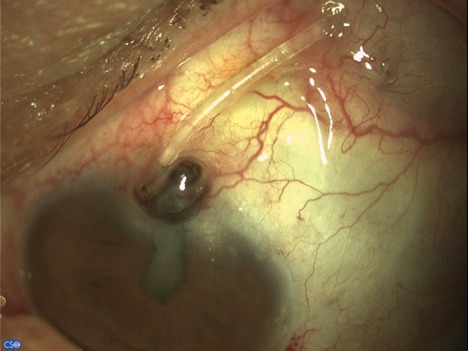
Tube exposure associated with corneal melting and iris prolapse

**Fig. 2:**
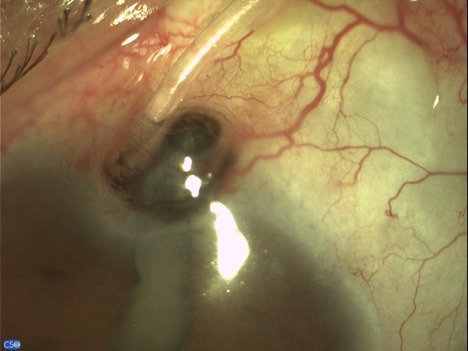
Tube exposure with conjunctiva scarring and epithelization of the tube

A 60-year-old patient arrived at the emergency room with tube exposure combined with corneal melting and iris prolapse (**Fig. 1**). The patient had a history of uveitic glaucoma that remained uncontrolled besides being on maximal topical and per os medical therapy and underwent Ahmed valve placement 6 months prior. The clinical examination revealed VA 1/10, IOP 7 mmHg with positive Seidel test and active leaking. Active inflammation with anterior chamber cells and flare of 2+ were observed. The entry point of the tube revealed corneal melting and neovascularization of the limbus with iris prolapse. The tube was completely exposed from the entry point up to the plate of the valve. The conjunctiva of the area was scarred and epithelization was noted surrounding the tube (**Fig. 2**).

The approach we decided to follow was to use one corneoscleral graft to repair the melted cornea and avoid further iris prolapse and a second scleral graft to cover the repositioned tube. 

**Fig. 3:**
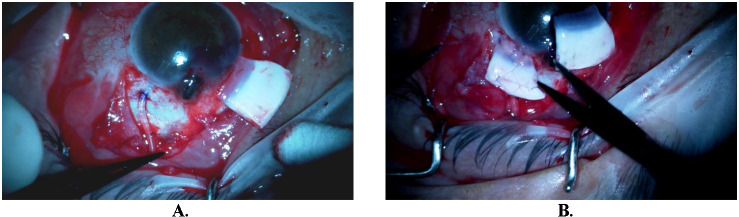
**A.** Tube’s new position 3 mm temporally to the previous entry point and 1.5 mm posterior to the limbus after conjunctival dissection; **B.** Limbal reconstruction with second alcohol preserved corneoscleral graft in specific orientation covering the protruding iris

Firstly, we carefully removed the tube from its position and dissected the conjunctiva surrounding the exposed tube under topical anesthesia. At that point, an effort to dislodge the protruding iris was done ab interno using the Viscoelastic cannula, but because excessive bleeding was noted and acute hyphema formation, we stopped and filled the anterior chamber with Viscoelastic to tamponade the bleeding and keep the anterior chamber formed. Upon completion of conjunctival dissection, with caution not to damage the tube in a wide area including the exposed area and the future entry point, cleaning and deepithelization of the melted cornea and the tube by application of alcohol 100% followed. Deepithelization consists of a crucial step to avoid future re-exposure and possibly epithelial ingrowth. Then, our attention was centered on the repositioning of the tube with caution not to violate the previously well-placed plate of the valve and its surrounding bleb. The tube was repositioned 3 mm temporally to the initial site and 1.5 mm posterior to the limbus and was covered using alcohol-preserved scleral allograft, soaked in ceftriaxone solution before placement, to avoid future exposure (**Fig. 3 A, B**).

**Fig. 4:**
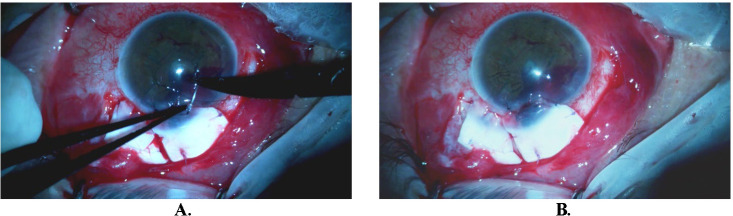
**A.** Corneal part of the second graft completely covers the melted cornea and prolapsed iris, secured in place by nylon 10-0 sutures, ensuring optimal stability and tissue integration; **B.** Grafts with specific orientation in their final position reinforcing the ophthalmic wall

We then focused on limbus reconstruction by suturing our second graft, an alcohol-preserved corneoscleral allograft, in a specific orientation. The graft was trimmed in such a fashion to reduce the chances of future dellen formation. The corneal aspect of the graft covered the melted cornea making sure that the prolapsed iris was completely covered and was secured using 10-0 nylon sutures. The scleral part strengthening the underlying sclera using vicryl sutures restored the anatomy of the area and the limbus (**Fig. 4 A, B**).

**Fig. 5:**
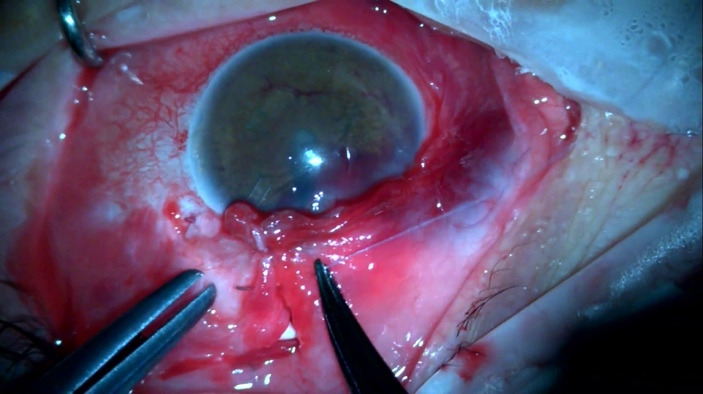
Conjunctival closure for both grafts achieved through manipulation of the fornix conjunctiva to the designated area. Vicryl 8-0 sutures were used to secure the closure in a watertight manner, ensuring effective wound healing and protection

Finally, we achieved conjunctival closure for both grafts by maneuvering fornix conjunctiva to the area and using vicryl 8-0 sutures to secure in a watertight fashion (**Fig. 5**).

## Results

**Fig. 6:**
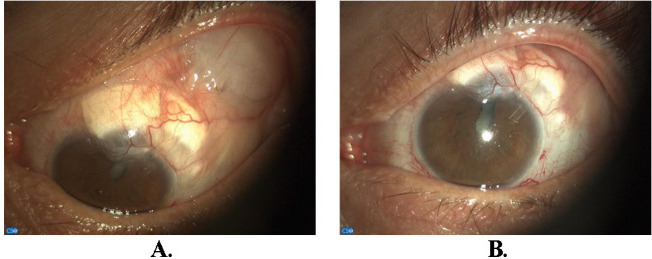
Limbal reconstruction with a well-positioned tube in the anterior chamber (**A.** downward gaze, **B.** primary position). Note the formed circumferential vessel suggesting adequate blood supply to the area and a smooth healing process

The postoperative course was smooth and the patient’s 6-month follow-up revealed an intact globe, without any area of active leaking, both grafts in place, good tube positioning without any exposure, and an in-target IOP of 14 mmHg (**Fig. 6 A, B**).

## Discussion

Ahmed valve implantation has been established as a suitable glaucoma surgery option for uveitic glaucoma patients not only because this glaucoma drainage device (GDD) reduces IOP and preserves visual acuity, but also because in almost all of the published studies, the complication rates are lower than other GDDs or trabeculectomy [**1**,**6**,**7**]. Ahmed valve placement may be the first choice for surgical intervention especially in patients with active inflammation or in those with high-risk factors for trabeculectomy failure [**8**,**9**]. Nevertheless, tube exposure is a serious complication because it represents a major risk factor for the development of late endophthalmitis due to the pathway for the organisms that the tube provides through the erosion of the conjunctiva. Additionally, the list of complications includes inflammation, hypotony, poor vision, and phthisis.

According to clinical studies, the young age and the presence of inflammation before tube exposure are the key factors that can cause a valve to be exposed out of its position [**10**]. The frequency of tube extrusion when the Ahmed device is implanted varies from 7% to 14,3% and the surgical intervention is of vital importance [**11**].

Surgical repair of these cases can be very demanding and requires surgical improvisation and prolonged surgical time. The application of corneoscleral allograft provides tensile strength and rigidity due to the thickness of the tissue and that is why it constitutes a great solution for the repair of the defects in the ocular wall [**12**]. In our case, corneal melting and iris prolapse were associated as a result of a combination of tube initial malpositioning and ocular inflammation making the case even more challenging and requiring in situ surgical improvisation. Literature remains very limited on how a surgeon should approach similar cases, which are the crucial tips and which are the missteps that should be avoided. In this case, we used multiple scleral/corneoscleral allografts in specific orientations and different sutures to reconstruct the damaged limbal area and restore the anatomy. Another crucial step is to try and preserve the valve, since in many of these cases it is a functioning drainage implant and effort should be made to save the implant when possible. One of the greatest challenges in similar cases remains the conjunctival closure since the tissue is very thin, in some cases nonexistent, and inflamed, making maneuvering very difficult and associated with excessive surgical bleeding. These are signs that may often be underdiagnosed preoperatively and lead the surgeon to underestimate the complexity of the surgery that will follow.

Regarding the clinical significance, published data on re-exposure of Glaucoma Drainage Devices is limited and it remains debatable on which should be the optimal approach for these cases [**13**,**14**]. Larger studies with a variety of different cases and long-term follow-up will be needed in the future to determine the ideal surgical technique and the best materials, both enduring and biocompatible, to be selected for complex cases like the one presented.

## Conclusion

Surgical repair of these cases can be very demanding and requires surgical improvisation and prolonged surgical time. Application of corneoscleral and scleral allograft provides tensile strength and rigidity due to the thickness of the tissue and that is why it constitutes a great solution for the repair of defects in the ocular wall. Because of the variety of different materials described in the literature, it is important to select the most appropriate material depending on the case, and in very complex cases, such as the one presented, combine them appropriately to achieve the best outcome.

**Conflict of Interest Statement**

The authors state no conflict of interest.

**Informed Consent and Human and Animal Rights Statement**

The accumulation of data was carried out with approval from our Institutional Review Board (IRB). Written consent to publish the case was not obtained. This report does not contain any personal identifying information. 

**Authorization for the use of human subjects**

Ethical approval: The research related to human use complies with all the relevant national regulations and institutional policies, as per the tenets of the Helsinki Declaration and has been approved by the review board of National and Kapodistrian University of Athens, 1st University Eye Clinic, G. Gennimatas General Hospital, Athens, Greece (RN:#20022321045, 27.03.2023).

**Acknowledgments**

None.

**Sources of Funding**

No funding or grant support.

**Disclosures**

None.
